# Supplementary material for: Deep Learning Classification of Canine Behavior Using a Single Collar-Mounted Accelerometer: Real-World Validation
Source: Animals (Basel). 2021 May 25;11(6):1549. doi: 10.3390/ani11061549 (PMC8228965; doi:10.3390/ani11061549)
Supplement: Supplementary file 1 [file animals-11-01549-s001.zip › animals-1190134-supplementary.pdf]

Supplementary Materials

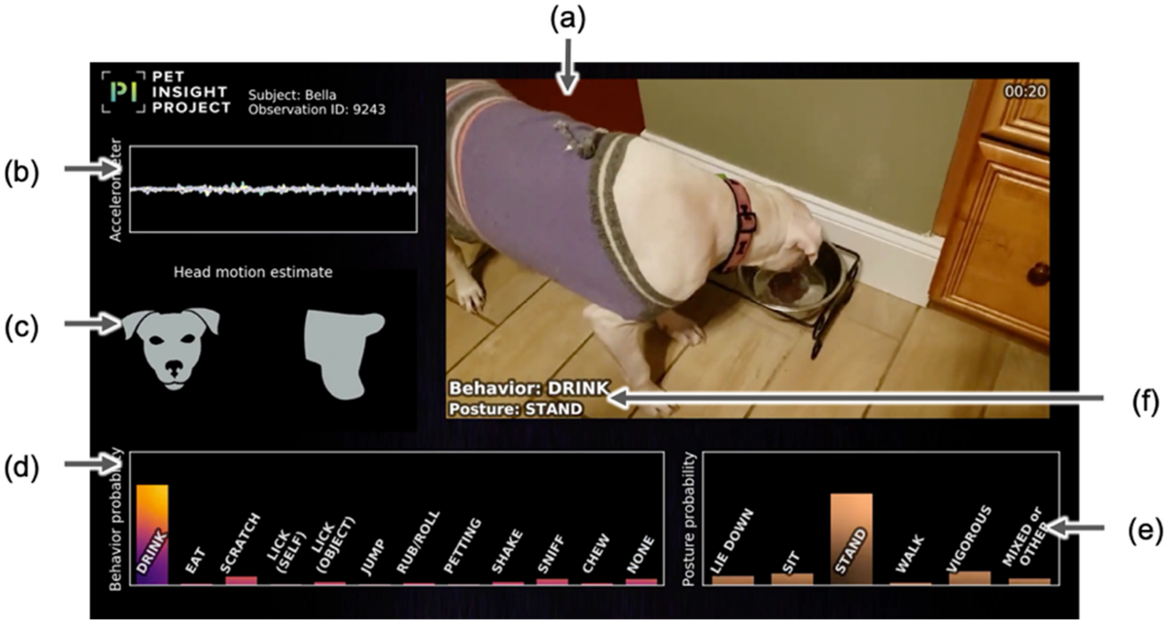

Figure S1: Guide to interpreting supplementary videos. The videos listed below follow the template in this frame capture. The source video file is played in an inset (a), and the synchronized accelerometer data is scrolled through the plot in (b). The head motion estimate produced by the algorithm (accounting for device position and orientation on the collar) is visualized in (c). The algorithm estimates the probability that various behaviors (d) and postures (e) are occurring, based solely on the accelerometer data (the video data is not seen or otherwise used by the algorithm). A small visual display of the most likely predicted class is superimposed on the inset (f).

Table S1: Index of supplementary videos

**Reason Included**	**Video**
Examples of eating- and drinking-related behaviors, including licking the empty bowl.	S1, S2, S3
Example of general behavior, including playing.	S4
Examples of dermatology-related behaviors: scratching, self-licking, rubbing, and shaking.	S5<br>S6
Classification of eating behavior while eating from a slow-feed bowl, which presents some challenges to the algorithms.	S7
